# Symptom Management Following Radioactive Iodine Treatment in Adults With Thyroid Cancer: Usability Evaluation and Proof-of-Concept Study

**DOI:** 10.2196/84151

**Published:** 2026-10-13

**Authors:** Alaina Carr, Angela M Jenkins, Valeria Gómez, Janine Alexandria Lorenzo, Ryan Moclair, James Pocchia, Devansh Gandhi, Kristen Miller, Kristi D Graves, Jacqueline Jonklaas

**Affiliations:** 1 Department of Oncology Georgetown University Medical Center Washington, DC United States; 2 Department of Biology College of Arts and Sciences Georgetown University Washington, DC United States; 3 National Center for Human Factors in Healthcare MedStar Health Research Institute Washington, DC United States; 4 Division of Endocrinology Georgetown University Washington, DC United States

**Keywords:** internet-based intervention, iodine radioisotopes, self-efficacy, survivorship, symptom burden, thyroid neoplasms, user-centered design

## Abstract

**Background:**

Thyroid cancer is often labeled a “good cancer” due to high survival rates. However, many patients diagnosed with differentiated thyroid cancer (DTC) treated with radioactive iodine (RAI) experience short- and long-term symptoms as a result of treatment. These include salivary dysfunction, dry mouth, dry eyes, and nausea. Despite these challenges, patients report limited knowledge and unmet informational needs regarding RAI symptoms and symptom management. Evidence-based, patient-centered education to support symptom management during DTC survivorship is needed. Our team developed Radioactive Iodine Support (RAI Support), a prototype of a 4-week web-based intervention for RAI symptom management among survivors of DTC.

**Objective:**

This study aimed to evaluate the usability of the RAI Support intervention among survivors of DTC who completed RAI treatment and assess preliminary effect sizes of change in RAI symptom burden, self-efficacy for managing chronic symptoms, informational needs, global physical and mental health, and health-related quality of life (HRQOL).

**Methods:**

We conducted a longitudinal usability testing study with 8 participants over a 4-week period. At baseline (T1), participants completed an online survey and a 90-minute virtual usability testing session. During usability testing sessions, study staff used a think-aloud protocol to elicit feedback on the content, language, and visual appeal of the prototype across 5 tasks. Participants also completed the System Usability Scale (SUS). Participants were encouraged to continue using the intervention over the 4-week period, during which use data were recorded. A follow-up after assessment (T2) was completed at the end of the 4-week period of home use. Survey outcomes included RAI symptom burden, self-efficacy for managing chronic symptoms, informational needs, global physical and mental health, and HRQOL.

**Results:**

Among the 8 survivors of DTC who had undergone RAI treatment (age: mean 45, SD 8.2 years), the mean SUS score was 88.1 (SD 8.7), indicating above-average usability. All participants effectively completed each task, with ease of use ranging from 100% to 87.5% and error rates ranging from 0% to 25%. Preliminary *r* effect size signals of change were found for improved self-efficacy, global mental health, and HRQOL (*r* values ranged from 0.31 to 0.45).

**Conclusions:**

Survivors of DTC reported above-average usability of the RAI Support prototype and completed each task successfully. Variability in error rates and ease of use suggests that refinements are needed to further optimize usability and patient engagement. RAI Support also shows preliminary promise in improving self-efficacy for symptom management, global mental health, and HRQOL among survivors of DTC. Findings support future pilot testing.

## Introduction

Thyroid cancer is frequently referred to as a “good cancer” due to its excellent 5-year survival rates and favorable prognosis [1-5]. However, this label often minimizes the lived experiences that many patients undergo. Radioactive iodine (RAI) treatment is not routinely indicated for all patients with thyroid cancer. Those who do receive RAI typically have undergone total or near-total thyroidectomy, after which postoperative risk stratification incorporating histopathology, staging, and imaging determines whether RAI is indicated for remnant ablation, adjuvant therapy, or treatment of known residual or recurrent disease [6]. Because RAI is used selectively based on these characteristics, those who do receive it often represent a subgroup with higher-risk cancer who correspondingly experience a longer course of surveillance and symptom management [6,7]. These patients report significant and persistent side effects such as salivary gland dysfunction, dry mouth, eye irritation, and nasal discomfort [1,8,9]. These symptoms can negatively impact quality of life, especially in the absence of coordinated, ongoing support [10,11]. Survivors of thyroid cancer also report lower health-related quality of life (HRQOL) than other groups of survivors of cancer [11,12], perhaps due, in part, to symptom burden [11]. Following RAI treatment, care coordination for symptom burden is often inconsistent across health care settings and typically involves multiple subspecialists, including endocrinologists, nuclear medicine physicians, oral health specialists, and otolaryngologists, which may contribute to fragmented follow-up care [10-13].

As cancer survivorship care remains fragmented and increasingly extends beyond traditional clinical settings, scalable strategies are needed to support patient engagement and symptom management throughout the continuum of care [14]. Digital health interventions have demonstrated potential for addressing gaps in survivorship care after primary treatment, particularly by enhancing access to timely support and empowering patients to manage chronic symptoms [15,16]. Despite the complexities of thyroid cancer survivorship care, we are unaware of any dedicated digital health tools to support symptom self-management after RAI treatment, presenting a critical gap in the continuum of care for this population.

We developed Radioactive Iodine Support (RAI Support) as a web-based intervention to fill this unmet need by providing tailored guidance and symptom support to survivors of thyroid cancer following RAI treatment. A number of systematic reviews have highlighted the facilitators and barriers to behavioral intervention adherence due to digital technology–related factors, including website usability ratings, ease of use, plain language, and connectivity [15-17]. One systematic review identified that low ease of use and connectivity issues were associated with 8% to 14% of intervention nonadherence [17]. The literature also highlights that the successful implementation of sustainable digital tools depends heavily on their usability, accessibility, and alignment with patient needs [18,19]. Yet, as with all digital interventions, optimal usability is a necessary component of sustained user engagement, which in turn relates to the efficacy of the digital tool. Human factors, such as interface design, ease of navigation, and relevance of content, play central roles in determining whether users adopt, engage with, and benefit from such tools [16]. Without attention to these factors, even well-designed interventions may fail to translate into real-world impact. Therefore, we conducted a usability and proof-of-concept study to (1) optimize the usability of the web-based intervention and (2) provide an initial exploration of whether RAI Support improves clinically significant end points, with the aim of addressing the complex needs of survivors of thyroid cancer in preparation for future pilot testing.

## Methods

### Study Design

From September to November 2024, we conducted usability testing in a single-arm longitudinal proof-of-concept study at an academic medical center in the mid-Atlantic United States. A sample size of 8 participants was selected for the usability evaluation, consistent with the study’s objective of identifying usability issues [20] with RAI Support and obtaining user feedback to inform intervention optimization rather than testing intervention efficacy.

### Recruitment and Setting

Adult patients were identified from medical record review and through a thyroid cancer nonprofit organization’s publicly available Facebook (Meta Platforms Inc) page, on which the study was advertised. The 2 sources for recruitment were intended to understand the spectrum of experiences of survivors of differentiated thyroid cancer (DTC), RAI symptom burden profiles, and website feedback from individuals across the United States who were treated across a variety of hospital settings.

Eligible patients met the following inclusion criteria: (1) age of ≥18 years; (2) able to provide informed consent in English; (3) diagnosed with DTC (papillary or follicular carcinoma) in the past 3 years; (4) received RAI treatment within the past 3 years; (5) access to a computer, tablet, or smartphone with internet connectivity; and (6) completion of the Radioiodine-6 screener [21] with a total score of ≥7.6 or a score of ≥3 on at least 1 out of the 3 RAI symptom subitems (mouth, eyes, or nasal). Exclusion criteria included non–English-speaking patients and individuals with cognitive, psychiatric, or hearing impairments that could limit their ability to consent or participate in the study.

Following institutional review board approval (#00005285), potential participants were contacted through introductory emails or mailed study flyers, followed by a Health Insurance Portability and Accountability Act (HIPAA)–compliant videoconference with research staff to assess eligibility through the completion of a screening questionnaire. Informed consent was obtained through a 15-minute telephone call or a HIPAA-compliant videoconference. Participants reviewed and signed the informed consent form electronically. A total of 8 patients diagnosed with DTC and treated with RAI within the past 3 years were enrolled.

### Imposter Participants

Our Facebook recruitment approach also led to 29 “imposter participants,” defined as individuals who provide false identities and information for the purpose of participating in research studies [22-24]. Imposter participants were identified by a combination of the following indicators: nonworking telephone numbers or telephone numbers for businesses, refusal to turn on their cameras (a requirement for usability participation), use of similar (yet unusual) email domains, and inconsistent internet connectivity.

To increase rigor and guard against imposter participants, study staff modified the screening procedure to include open-ended questions about the name of the hospital or clinic setting in which participants received treatment. We then ended Facebook recruitment to reduce the likelihood of additional imposter participants. Of note, imposter participants were not included in the final data analysis.

### Data Collection

Participants completed baseline and postintervention surveys via REDCap (Vanderbilt University) [25] (for details, refer to the Measures section).

Study staff trained in qualitative research methods developed the semistructured usability testing interview guides (including author AC, a female psychologist, author KM, a female human factors scientist, and a nonauthor, female research and human factors scientist). Interviews lasted approximately 90 minutes.

Study staff (authors) trained in qualitative research conducted the interviews: AMJ (a female research assistant), JP (a male research assistant), and RM (a male research assistant). A second member of the research team (JAL, a female research assistant, and JP), trained in qualitative data research, took detailed notes during the interviews to track usability task outcomes.

Study staff used a think-aloud method to prompt participants to provide feedback as they navigated key website elements, including the log-in process, home page, welcome video, symptoms page, salivary symptom management page, and resource page. Feedback focused on content clarity, visual appeal, and ease of navigation (Figure 1). All recorded usability testing interviews were transcribed verbatim and deidentified prior to data analysis. At the end of the usability testing interviews, participants completed a System Usability Scale (SUS) questionnaire [26] via REDCap. Participants were then asked to continue using RAI Support for 4 weeks. At the conclusion of the 4 weeks, they completed a follow-up questionnaire.

**Figure 1:**
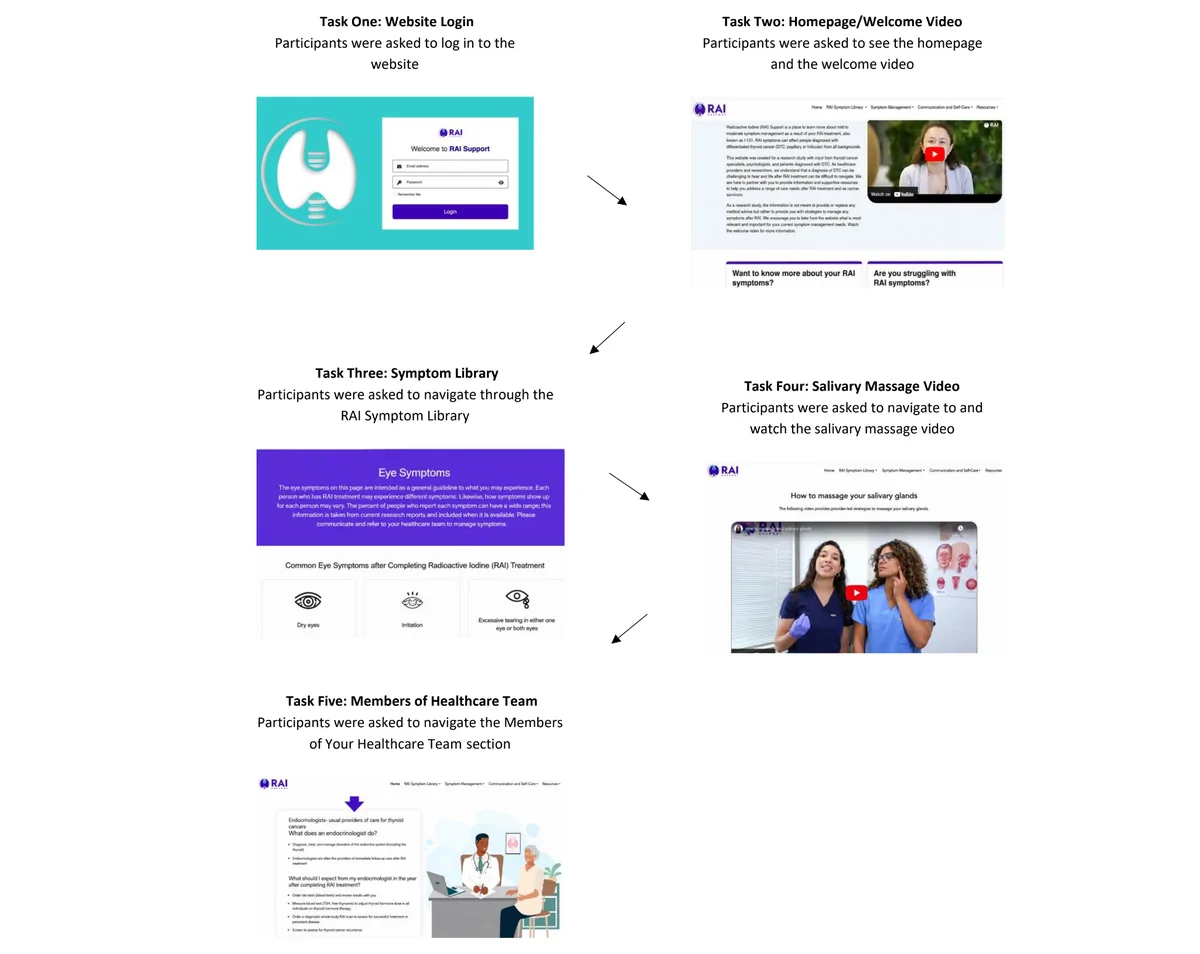
Screenshots of web-based Radioactive Iodine Support (RAI Support) prototype.

### Intervention

RAI Support has been previously described [9]. RAI Support is a theoretically driven web-based intervention to support self-management of RAI symptoms among survivors of DTC. RAI Support is informed by social cognitive theory [27] and was developed following person-based design principles, with survivors of DTC and health care providers (endocrinologists, endocrine surgeons, nuclear medicine providers, registered oncology dietitians, social workers, and psycho-oncologists) as integral members of the intervention development process [28]. The web-based intervention is asynchronous and consists of content related to education on RAI symptoms**;** brief provider-led videos on symptom management strategies for mild to moderate symptoms**;** communication and self-care, including mindfulness exercises**;** and DTC survivorship resources. Website programming was produced by DreamSoft4u, Pvt. Ltd [29].

### Measures

#### Usability Evaluation Outcome Measures: SUS

The SUS is a 10-item questionnaire that assesses patients’ perceived usability of a system [26]. Items are rated on a 5-point Likert scale (1=“Strongly disagree” to 5=“Strongly agree”), with composite scores ranging from 0 to 100. A SUS score >68 represents above-average usability [26].

Effectiveness is defined as the success rate or the degree to which a participant can successfully achieve their goal in using the system. For the present study, effectiveness is assessed dichotomously (yes or no) to indicate the ability to complete a task.

Ease of use is the degree to which a participant can complete a task on their very first attempt without training. For the present study, ease of use is assessed dichotomously (yes or no) and refers to the ability to complete a task the first time.

Error rate is the degree to which the interaction between the participant and the system is smooth and/or the type of mistakes a participant makes while completing each task. For the present study, error rate is assessed on a 4-point Likert scale (1=“cosmetic or visual error,” 2=“minor error or user recovers quickly,” 3=“major error or user recovers with difficulty,” and 4=“catastrophic error or system crashes”), with higher scores indicating greater severity of error.

#### Intervention Outcome Measures

##### Salivary, Lacrimal, and Nasal Measure

The salivary, lacrimal, and nasal (SALANS) [8] measure consists of 33-items and assesses SALANS symptoms among patients with DTC after RAI treatment on a 5-point Likert scale (0=“Not at all” to 4=“Very much”). The scale has 6 subscales (sialadenitis, taste, xerostomia, dry eyes, epiphora, and nasal). Higher scores indicate greater symptom burden. The internal consistency α coefficient for the current sample is 0.98.

##### Functional Assessment of Cancer Therapy-General-7 Item

The Functional Assessment of Cancer Therapy-General-7 Item (FACT-G7) [30] is a 7-item questionnaire that assesses physical, emotional, and functional well-being of adult oncology patients. Each item is rated on a 5-point Likert scale (0=“Not at all” to 4=“Very much”). Scores range from 0 to 28, with higher total scores indicating greater HRQOL. The α coefficient for the current sample is 0.88.

##### Patient-Reported Outcomes Measurement Information System Scale

The Patient-Reported Outcomes Measurement Information System (PROMIS) Scale version 1.2 Global Health is a 4-item assessment of physical and mental health measured on a 5-point Likert scale [31]. Raw summed scores range from 4 to 20 and are converted to T-scores, with a mean of 50 and an SD of 10. A higher T-score indicates greater physical and mental health. The α coefficient for the current sample is 0.92.

##### PROMIS Self-Efficacy for Managing Chronic Conditions–Managing Symptoms

The PROMIS Self-efficacy for Managing Chronic Conditions–Managing Symptoms is a 28-item questionnaire that evaluates physical, mental, and social health in individuals with chronic conditions, with the T-score (mean 50, SD 10) representing the final score [32]. A higher score indicates higher self-efficacy. The α coefficient for the current sample is 0.96.

##### Informational Needs Questionnaire for DTC

The Informational Needs Questionnaire for DTC (INQ-DTC) contains 33 items and assesses informational needs of patients after RAI treatment [33]. Items are measured on a 5-point Likert scale (1=“Not at all” to 5=“Very”). Higher total scores indicate greater informational needs. The α coefficient for the current sample is 0.93.

### Exit Questionnaire

Participants completed an exit questionnaire to assess their perceptions of the helpfulness of components of the RAI Support intervention, likelihood of recommending the program to survivors of DTC, satisfaction with intervention length, and overall satisfaction with the program. Responses were assessed using 10-point Likert scales for the majority of items.

### Intervention Back-End Use Data

Intervention back-end use data were captured through the RAI Support back-end system. Metrics captured included frequency of log-ins and number of website pages visited. Use data were exported in aggregate form for analysis to complement self-report measures of intervention engagement.

### Data Analysis

A mixed methods approach was used, incorporating quantitative data from questionnaires, usability logs, and the web-based intervention back-end system, as well as qualitative data from usability interviews for analysis. Descriptive statistics were used to summarize the demographic and clinical characteristics and web-based intervention use of participants. Signals of change in outcomes of interest were evaluated using within-group effect sizes calculated with the Rosenthal effect size formula (*r*=Z/√N), where *Z* is the standardized Wilcoxon signed-rank test statistic and *N* is the number of paired observations [34,35]. Cohen guidelines for *r* effect sizes were used and defined as “small” for *r*=0.1; “medium” for *r*=0.3; “large” for *r*=0.5 [35]. Signed effect sizes were reported to indicate the direction of change, with directionality defined based on follow-up minus baseline. Positive values indicate increases from baseline, whereas negative values indicate decreases.

Content analysis was used to analyze the usability testing interviews [36,37]. To gain an initial understanding of the content, 3 authors (AC, AMJ, and JAL) independently reviewed the interview transcripts and met biweekly to identify and refine preliminary codes and examine data discrepancies until team resolution. Coding was conducted using ATLAS.ti Scientific Software Development GmbH (version 8.15.3; Lumivero LLC). Three authors (AC, AMJ, and JAL) collaboratively developed a codebook, consolidating initial codes into broader categories through iterative discussion. These categories were further synthesized into major themes and subthemes to capture key patterns in participant feedback regarding the context underlying usability issues and potential methods for optimization. Thematic saturation [38] was operationally defined as the point at which review of interviews did not identify new themes or subthemes relevant to usability. Using this approach, no new themes or subthemes emerged from the final interview data, indicating thematic saturation within the scope of the usability evaluation.

### Ethical Considerations

Ethics approval was obtained in accordance with the principles of the Declaration of Helsinki. The protocol was reviewed and approved by the Georgetown University Institutional Review Board (#00005285). All participants provided signed informed consent. Study data were deidentified prior to data analysis. Participants were compensated with a US $50 gift card for study participation.

## Results

### Recruitment

A total of 76 individuals were assessed for eligibility (Figure 2). A total of 47 (61.8%) individuals contacted study staff in response to Thyroid Cancer Survivors’ Association Inc (ThyCa) Facebook advertisements, and 29 (38.2%) individuals were screened for eligibility from medical records. In total, 24 participants declined study participation: 19 were not interested and 5 were too busy. Overall, 64 individuals were excluded, including 24 who declined and 40 who were ineligible for the following reasons: 3 were diagnosed with DTC >3 years ago, 1 received RAI >3 years ago, 1 did not receive RAI, 1 was not an English speaker, 29 were imposter participants, and 5 did not meet screening criteria. Among those who were eligible, 12 participants consented to the study.

Of the total sample of consented participants (n=12), 1 was lost to follow-up and 3 withdrew, leaving a final sample of 8 consented participants who completed baseline, usability testing, and postassessment procedures. In the final study sample (N=8), the average age of participants was 45 (SD 8.2; range 35-61) years. In terms of clinical characteristics, the average times since diagnosis and RAI treatment were 1.2 (SD 1.1) and 0.6 (SD 0.4) years, respectively. Most participants were diagnosed with papillary thyroid cancer (7/8, 87.5%), and most received an RAI dose of 100 to 150 mCi (n=6, 75%). See Table 1 for demographics and clinical characteristics.

**Figure 2:**
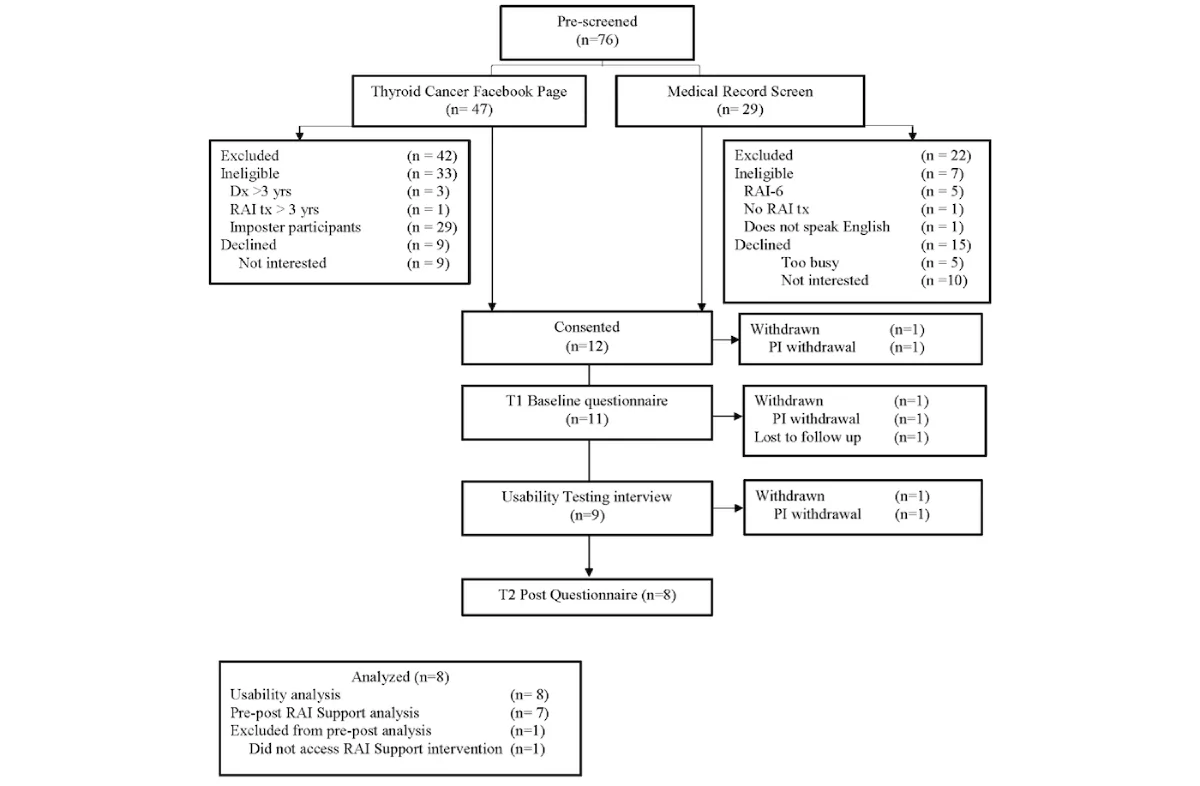
Study flow diagram. PI: principal investigator; RAI: radioactive iodine.

**Table 1:** Demographics and clinical characteristics of participants (N=8).

Variables		Participants
Age (years), mean (SD; range)		45 (8.2; 35-61)
**Sex, n (%)**		
	Male	1 (12.5)
	Female	7 (87.5)
**Race or ethnicity, n (%)**		
	Asian	2 (25)
	Black or African American	1 (12.5)
	White	5 (62.5)
**Relationship status, n (%)**		
	Married	4 (50)
	Divorced	1 (12.5)
	Never married	3 (37.5)
**Education, n (%)**		
	Associate degree or some college	1 (12.5)
	College degree or higher	7 (87.5)
**Employment status, n (%)**		
	Employed (full-time)	5 (62.5)
	Employed (part-time)	1 (12.5)
	Self-employed	1 (12.5)
	Unemployed	1 (12.5)
Health insurance (private), n (%)		8 (100)
**Household income (US $), n (%)**		
	No income	1 (12.5)
	100,000-149,999	2 (25)
	150,000-199,999	1 (12.5)
	≥200,000	4 (50)
**Disability, n (%)**		
	Yes	1 (12.5)
	No	7 (87.5)
Time since diagnosis (years), mean (SD; range)		1.2 (1.1; 0.5-3.0)
Time since RAI^a^ treatment (years), mean (SD; range)		0.64 (0.4; 0.13-1.26)
**Cancer histology, n (%)**		
	Papillary	7 (87.5)
	Do not know	1 (12.5)
**RAI dose (millicuries), n (%)**		
	30-75	1 (12.5)
	76-100	1 (12.5)
	101-150	6 (75)

^a^RAI: radioactive iodine.

### Usability Evaluation

Eight participants completed usability testing sessions. The mean SUS score was 88.1 (SD 8.7), indicating above-average usability. All participants effectively completed each task. Ease of use, as measured by completion of a task on the first attempt, ranged from 100% to 87.5%, and error rates ranged from 0% to 25%. Participants rated the clarity of the welcome page (mean 4.3, SD 0.8) and the welcome video (mean 5.0, SD 0; task 1), indicating that the page was perceived as clear and the video as very clear. Mobile device screen sharing blocked the visuals needed to complete tasks 3 (RAI symptom library) and 4 (health care provider–led salivary gland massage video) for 1 participant. When the screen sharing issue was resolved with prompting from study staff, ease of use for this participant increased to 100% for task 5. Refer to Table 2 for an overview of the usability outcome metrics, including error rates by task.

**Table 2:** Usability evaluation outcomes (N=8).

Tasks				Participants
**Task 1: website log in**				
	Effectiveness			8 (100)
	Ease of use			8 (100)
	Error: none			8 (100)
**Task 2: home page and welcome video**				
	Effectiveness			8 (100)
	Ease of use			8 (100)
	Error: none			8 (100)
**Task 3: radioactive iodine symptom library**				
	Effectiveness			8 (100)
	Ease of use			7 (87.5)
	**Error**			
		None	6 (75)	
		Cosmetic	1 (12.5)	
		Minor	1 (12.5)	
**Task 4: health care provider–led salivary massage video**				
	Effectiveness			8 (100)
	Ease of use			7 (87.5)
	**Error**			
		None	6 (75)	
		Cosmetic	1 (12.5)	
		Minor	1 (12.5)	
**Task 5: members of your health care team**				
	Effectiveness			8 (100)
	Ease of use			8 (100)
	**Error**			
		None	7 (87.5)	
		Cosmetic	0 (0)	
		Minor	1 (12.5)	

### User Testing Feedback

Participants reported that they found the website content “informative,” “helpful,” and that they would recommend the website to someone else experiencing RAI symptoms. User testing feedback provided insight into barriers to user engagement, contextual information about the root causes of barriers to user engagement with the prototype, and areas for improvement. Overall, participants highlighted navigation across website pages as a primary area for improvement. Participants noted challenges in locating specific content and suggested adding hyperlinks to facilitate navigation. Participants also reported being timed out of the website and suggested increasing the session idle time. Example suggestions for website improvement and refinement are illustrated in Table 3.

**Table 3:** User testing feedback.

Domains		Exemplar quotes	Refinements
**Navigation (all elements related to moving between website pages and additional functions to enhance user engagement with the website)**			
	Hyperlinks	“There should be like more of a connection between and leading you where to go. Or if you want to learn more about this, ‘Go here.’ I wanted to be guided to something more, instead of having to go look for it.” [PID^a^ 16] “I think, clicking to the solution. A direct click might have been beneficial.” [PID 58]	Incorporation of hyperlinks to facilitate user navigation within the system
	Session time-out	“Let’s see. I have to sign back in...Now, that might be something want to look into.” [PID 59] “Oops got logged out...Look at it logged out again.” [PID 16]	Increase in the session idle time
Design (reference to the visual appeal, length, formatting, or other design elements of the web-based platform, regardless of task or section of website)		“I think the fonts size has to be a little bit more uniform.” [PID 49] “Making it a less medicalized and more down to earth like you’re talking more to an actual human being instead of somebody specializes in it [thyroid cancer].” [PID 52] “It’s just three paragraphs. When you use bullets where it’s quick that makes for a better, cleaner, read.” [PID 59]	Inclusion of bullet points to ease readability and understanding of complex information
Features: welcome page or video (area for refinement to acclimate the user to key features of the intervention)		“I didn’t know if the [welcome] video was going to lead into more interactive things or showing what maybe clicking around or how to use the site.” [PID 58] “I don’t know if you want to include the navigation here as an additional video.” [PID 65]	Refinement of the welcome video to include website navigation instructions
Features: interactive (areas for refinement to reduce the static nature of the intervention)		“Graphic visuals, pop ups or things that move across the screen. Just so, it’s not like quite as static and just information sitting there. Make a little more flash to it, or activity.” [PID 16] “This is not static content that you’re presenting... And when I hover on top of this card, perhaps you can then pop that information out, or pop it as a mobile dialogue, or expand on the key concept, on this one box.” [PID 65]	Incorporation of interactive features to enhance user engagement

^a^PID: participant ID.

### Exit Questionnaire and Intervention Back-End Use Outcomes

Participant scores on the exit questionnaire (n=7) indicate moderate to high ratings of helpfulness for the website videos: managing dry mouth (mean 7.14, SD 2.11), changes in taste (mean 6.5, SD 2.65), and relaxation and mindfulness (mean 6.83, SD 1.94). Back-end use data indicate that, on average, 6 (85.7%) of the 7 website users engaged with 3 out of the 4 content sections during the 4-week home intervention period. Refer to Multimedia Appendices 1 and 2 for the participant exit questionnaire and back-end metrics.

### Preliminary Intervention Outcomes

Preliminary effect size signals of change were calculated for participants who completed baseline and postassessment measures (n=7). The distributions of scores on outcomes of interest are presented in Table 4. Overall, preliminary effect size signals of change were found in self-efficacy (*r*=0.32), global mental health (*r*=0.31), and HRQOL (*r*=0.45), with positive effect sizes indicating increases from baseline. A small negative effect size was observed for RAI informational needs (*r*=−0.15), indicating a favorable decrease in informational needs from baseline. RAI symptom burden and psychosocial informational needs remained relatively stable throughout the study, and global physical health decreased across study time points.

**Table 4:** Signals of change from baseline to postintervention assessment with RAI Support (N=7).

Measures			Baseline assessment		Postintervention assessment		Changes in mean from baseline		*r* effect sizes^a^
RAI^b^ symptom burden^c^, mean (SD; range)			62.3 (38.7; 35-148)		63.0 (36.6; 38-144)		+0.7		+0.03
Self-efficacy, mean (SD; range)			40.6 (5.8; 31.4-50.3)		44.2 (12.2; 30.6-69.5)		+3.6		+0.32
**Informational needs** **, mean (SD; range)**									
	RAI	28.0 (2.2; 24-30)		25.7 (5.8; 14-30)		−2.3		−0.15	
	Psychosocial	19.4 (5.7; 10-25)		19.1 (5.2; 10-25)		−0.3		0	
Global mental health, mean T-score (SE; range)			40.5 (3.45; 26.1-56.7)		42.5 (3.68; 21.3-48.8)		+1.9		+0.31
Global physical health, mean T-score (SE; range)			43.9 (4.11; 19.7-50.7)		42.7 (3.94; 19.7-50.7)		−1.2		−0.55
HRQOL, mean (SD; range)			15.0 (7.3; 0-22)		15.9 (7.5; 1-23)		+0.9		+0.45

^a^Cohen *r* effect size: 0.1=“small,” 0.3=“medium,” and 0.5=“large.”

^b^RAI: radioactive iodine.

^c^RAI symptom burden is measured by the salivary, lacrimal, and nasal (SALANS) measure.

## Discussion

### Principal Results

Findings from this study add to the literature by showing high usability and preliminary proof-of-concept data for a website designed to support symptom management following RAI treatment among patients with DTC. In addition, this is among the first studies to evaluate the usability of a web-based intervention for symptom management in an understudied population of survivors of DTC. Overall, participants found the RAI Support intervention to be informative and the health care provider–led videos to be helpful. Participant feedback identified user-centered areas to enhance the intervention’s ease of use, including navigation across webpages and interactive features to foster user engagement. Future adequately powered studies should further examine temporal patterns and predictors of intervention engagement, including whether sustained or repeated use varies by baseline RAI symptom burden, clinical characteristics, or recruitment source, to better understand for whom and under what circumstances the RAI Support intervention is most engaging. These focal points are consistent with broader gaps identified in the implementation literature on digital health interventions in thyroid cancer care. A recent scoping review of digital health interventions in thyroid cancer care synthesized intervention characteristics, effectiveness, and uptake, as well as reported barriers and facilitators, and identified few interventions focused on symptom self-management following primary treatment [39], highlighting the limited availability of digital approaches addressing this need. Within this context, among participants with complete pre-post RAI intervention data, we found evidence of small to medium clinically meaningful changes in self-efficacy for managing chronic symptoms, RAI informational needs, global mental health, and HRQOL. These findings support the potential of digital interventions such as RAI Support as a valuable and feasible approach to address symptom management needs, with further research and refinement likely to enhance their utility.

Results did not support RAI Support’s preliminary effect size signals of change on RAI symptom burden, informational needs for personal growth, and global physical health. One explanation for these findings is that the RAI Support intervention is intended to provide noncurative symptom management strategies, which may not eliminate the persistent nature of the frequency and severity of RAI symptoms [40-42]. Additionally, these data did not capture information on common medical comorbidities among survivors of DTC such as myocardial infarction, osteoporosis, hyperthyroidism, and hypothyroidism [10,11], which may explain the pre-post differences in PROMIS Global Physical Health scale scores in the sample. Future studies with larger samples that capture common medical comorbidities are warranted to further evaluate these outcomes. Another explanation for these findings is that the static informational content of the website may not fully address the range of patient needs. Future research could explore more integrative and interactive features, such as direct messaging with health care professionals or cancer-specific conversational agents to provide tailored guidance and deeper patient connection [43,44]. Recent interventions using a range of web-based elements yielded a reduction in chemotherapy-related side effects and improved effectiveness in self-care behaviors in patients with cancer [43,44]. These findings also contribute to a broader shift toward risk-adapted, digitally enabled thyroid cancer care. Advances in molecular testing and AI-assisted cytopathology are refining the diagnostic evaluation of indeterminate nodules, while biomarker approaches are being examined to refine selection for RAI [45-47]. Together, these changes further underscore the potential value of scalable digital survivorship interventions, such as RAI Support, for addressing persistent symptoms and supporting patients throughout posttreatment care.

### Limitations

There are several limitations of this study. Despite recruitment efforts through a publicly available thyroid cancer nonprofit Facebook page, most participants were recruited based on purposeful sampling from electronic medical record review. This led to participants in the study being relatively similar, with the majority being female, well educated, and from higher-income backgrounds (>US $200,000 per year). Although DTC does predominantly affect female individuals, having the preponderance of study participants come from such analogous backgrounds limits the generalizability of the study to larger and more diverse DTC populations. Usability and acceptability need to be confirmed in patients with broader socioeconomic, educational, and digital literacy backgrounds before implementation.

The small sample size of the study was appropriate to address the aims of the usability evaluation and ORBIT (Obesity-Related Behavioral Intervention Trials) model proof-of-concept testing, which focused on identifying preliminary effect size signals of change [20,48]. However, this limited sample size and the absence of a comparison condition further limit the generalizability of the findings and do not allow analyses of statistical differences or preliminary intervention effects. The study also covered a short time frame for participants, with user engagement and clinical outcomes tracked only over a 4-week period. This limited interval of analysis prevents a full understanding of the long-term benefits of the intervention for patients after RAI treatment.

An additional limitation is that the study encountered 29 imposter participants. Although those individuals were excluded, this recruitment issue is relevant to feasibility, recruitment, and generalizability of remote usability testing procedures and should be interpreted with caution [20,48]. The potential influence of outlier scores is another limiting factor, such as for PROMIS Global Physical Health, particularly given the small sample size. Future pilot feasibility trials should incorporate larger participant samples from different geographic areas, participants with varying intervals since RAI treatment, and a control condition to allow more rigorous evaluation of the RAI Support intervention.

### Conclusions

This study demonstrated a positive usability testing evaluation of the web-based RAI Support prototype for RAI symptom management among survivors of DTC, a population that has received limited survivorship-focused attention. The study also identified preliminary effect size signals of change in self-efficacy in survivors of DTC, global mental health, and HRQOL. Ongoing research has incorporated the patient-recommended refinements and will examine the feasibility and acceptability of the RAI Support intervention through a pilot randomized controlled trial.

## Appendix

Multimedia Appendix 1 Exit questionnaire summary after 4 weeks of at-home Radioactive Iodine Support (RAI Support) use.

Multimedia Appendix 2 Back-end log-in data from 4 weeks of at-home Radioactive Iodine Support RAI (RAI Support) intervention use.

## Abbreviations

DTC: differentiated thyroid cancer
FACT-G7: Functional Assessment of Cancer Therapy-General-7 Item
HIPAA: Health Insurance Portability and Accountability Act
HRQOL: health-related quality of life
INQ-DTC: Informational Needs Questionnaire for Differentiated Thyroid Cancer
ORBIT: Obesity-Related Behavioral Intervention Trials
PROMIS: Patient-Reported Outcomes Measurement Information System
RAI Support: Radioactive Iodine Support
RAI: radioactive iodine
SALANS: salivary, lacrimal, and nasal
SUS: System Usability Scale
ThyCa: Thyroid Cancer Survivors’ Association Inc

## Data Availability

The datasets generated or analyzed during this study are not publicly available due to ethical considerations related to the protection of participant confidentiality, given the small sample size in this study, and in accordance with the guidance of the Health Insurance Portability and Accountability Act (HIPAA) and the consent process but are available from the corresponding author on reasonable request.
