# Supplementary material for: Symptom Management Following Radioactive Iodine Treatment in Adults With Thyroid Cancer: Usability Evaluation and Proof-of-Concept Study
Source: JMIR Cancer. 2026 Oct 13;12:e84151. doi: 10.2196/84151 (PMC13714524; doi:10.2196/84151)
Supplement: Supplementary file 1 [file cancer_v12i1e84151_app1.docx]

## **Multimedia Appendix 1**

**Table 1.** Exit questionnaire summary after four weeks of participant RAI Support at-home use.

| **Question** | **N=8**  **N (%) or M (SD; range)** |
| --- | --- |
| Did you find four weeks to be too much time to use the RAI Support website?^a^ | 5.625 (3.20; 0-10) |
| How helpful were the RAI symptom management website?^b^ | 6.25 (3.45; 1-10) |
| Did you watch the video "Nutrition strategies to reduce dry mouth symptoms"?, Yes | 7 (87.5) |
| How helpful was the video "Nutrition strategies to reduce dry mouth symptoms?”^b^ | 7.14 (2.11; 4-10) |
| Did you watch the nutrition video, "Nutrition Strategies for Changes in Taste?”, Yes | 4 (50) |
| How helpful was the video "Nutrition Strategies for Changes in Taste?”^b^ | 6.5 (2.65; 4-10) |
| Did you watch the video "What is Mindfulness and Relaxation? How Does It Relate to RAI Treatment and Thyroid Cancer?", Yes | 6 (75) |
| How helpful as the video "What is Mindfulness and Relaxation? How Does It Relate to RAI Treatment and Thyroid Cancer?"^b^ | 6.83 (1.94; 5-10) |
| Did you watch the video "How Can I Use the Mindfulness and Relaxation Audio Recordings?", Yes | 5 (62.5) |
| How helpful was the video "How Can I Use the Mindfulness and Relaxation Audio Recordings?"^b^ | 7.2 (1.92; 5-10) |
| Did you listen to the relaxation and mindfulness audio recordings?, Yes | 4 (50) |
| How was the length of the relaxation and audio recordings (between 8 to 19 minutes)? |  |
| Too long | 0 (0) |
| Just right | 4 (100) |
| Too short | 0 (0) |
| How helpful were the mindfulness/relaxation recordings?^c^ |  |
| Resting with the breath | 1.67 (0.577; 1-2) |
| Making peace with your body | 1.67 (0.577; 1-2) |
| The expanding circle | 3.50 (0.707; 3-4) |
| Numbers in the sand | 4.50 (0.707; 4-5) |
| Changing the channel | 3.67 (1.15; 3-5) |
| Self-Reiki | 4.20 (1.79; 2-6) |
| Would you recommend this website to someone experiencing RAI symptoms?^d^ | 7.63 (2.77; 3-10) |

^a^1 = Not at All, 10 = Very Much

^b^1 = Not Helpful at All, 5 = Somewhat Helpful, 10 = Very Helpful

^c^1 = Most Helpful, 6 = Least Helpful

^d^ 1= Not at all, 10= Yes, definitely
