# Supplementary material for: Symptom Management Following Radioactive Iodine Treatment in Adults With Thyroid Cancer: Usability Evaluation and Proof-of-Concept Study
Source: JMIR Cancer. 2026 Oct 13;12:e84151. doi: 10.2196/84151 (PMC13714524; doi:10.2196/84151)
Supplement: Supplementary file 2 [file cancer_v12i1e84151_app2.docx]

## **Multimedia Appendix 2**

**Figure One.** Backend login data from the four weeks of at-home RAI Support intervention use. (a) Weekly participant login data; (b-e) section-based RAI support login data: (b) RAI library sections; (c) symptom management sections; (d) communication and self-care sections; (e) resources sections.
